# Supplementary material for: DNA Methylation and Prospects for Predicting the Therapeutic Effect of Neoadjuvant Chemotherapy for Triple-Negative and Luminal B Breast Cancer
Source: Cancers (Basel). 2023 Mar 6;15(5):1630. doi: 10.3390/cancers15051630 (PMC10001080; doi:10.3390/cancers15051630)
Supplement: Supplementary file 1 [file cancers-15-01630-s001.zip › cancers-2188016-supplementary.pdf]

Supplementary material

List of differentially methylated genes in TN breast cancer samples with different response to NACT

ABCA17P, ABCA3, ADAMTS2, ADAP1, AKT3, ALOXE3, ATP8B2, B4GALT7, BCL2L1, BEGAIN, BMP2, BMP3, BMPER, C1orf86, CAPN2, CCDC69, CD8BP, CDC34, CDO1, CELF2, CHRNA7, CLDND1, CLEC14A, CLIP3, CNTN4, COL4A1, COL4A2, CRIP1, CRTC3, CSF3R, DCHS1, DLEU2, DMRT2, DMTN, DNAH10, DNAH3, DPYS, EBF4, EHMT1, ELAVL3, EPHA1, EPHA1-AS1, ESPN, EXOC6, FAM149A, FAM163A, FAM69C, FBN1, FBXW7, FMNL1, FOXR1, FYTTD1, GABRA5, GALR2, GAS6, GAS6-AS2, GATA6, GCOM1, GFRA1, GIPR, GLT1D1, GMDS, GNG10, GP5, GPC2, GPC4, GRIK1, GRIN1, HLA-AS1, HLX, HOXC13, HOXC13-AS, HPS1, HRAS, HUNK, IFNL3, INTS4L1, IRF4, KBTBD11, KCNIP2, KCNK12, KCNK17, KIAA0226, KIAA0930, KIAA1462, KLHL30, KRT8, LAMA3, LGALSL, LIMS2, LRCH2, LRRC27, LRRC56, LYPD6, MACROD1, MATK, MCOLN3, MFNG, MIR4453, MLC1, MPV17L, MRPL20, MT1G, MT1H, MYO15B, MYZAP, NANOS3, NEK4P2, NID2, NKX2-2, NKX2-2-AS1, NOTUM, NTN1, NTRK1, NUMBL, OR7E14P, OTUB1, PALM, PAX9, PDSS1, PHLDA2, PLEKHA7, PNCK, POLR2M, PPP4R1, PRR25, PTGER2, PTPRN, RAB34, RAB9A, RALA, RCE1, RFX1, RGS7, RN7SL657P, RNA5SP488, RNF128, RPH3AL, RPL23A, SCO2, SEMA3B, SEMA6D, SFMBT1, SFRP2, SHH, SMARCD3, SNORD42B, SOX21, SOX21-AS1, SPNS2, SRP68, SYNGR3, SYT12, TBCC, TCERG1, TERT, TLX3, TMEM200B, TNFRSF25, TNNI2, TOB1, TOB1-AS1, TRIP13, TUBB6, TWIST2, UBL4A, UBTF, UNCX, WDR13, ZNF385A, ZNF747), ACTL6B, ADAMTSL5, ADCY4, AGAP3, AJAP1, ALDH3B1, ALOX12, ANKRD23, ARHGAP8, ASPDH, B3GNTL1, BCAS2, BHLHA9, BMS1P18, BNC1, CACNA1H, CAPN15, CD46P1, CELF4, CHTF18, COL18A1-AS2, CPXM2, CPZ, CR1L, DBN1, DOCK1, EBF1, EDA, EFNB1, EMILIN1, EML2, ENTPD8, EPO, EVPL, FAM132B, FAM228A, FEV, FGF13, FGF13-AS1, FTH1P19, FZD10, FZD10-AS1, GBX1, GFM2, GPR143, H2BFM, HEYL, HMHA1, HMX2, HOXA9, HTATSF1, IFI27, INTS1, IRF7, ISLR2, JOSD2, KCNA5, KCNQ1OT1, KLHL15, LHX3, LINC00354, LINC01006, LYSMD2, MAMDC2, MAPK8IP2, MEG3, MEGF8, MIR4478, MIR5587, MOSPD1, MTMR1, MXRA5, NARFL, NAT9, NSA2, NSUN5, NSUN5P1, OLFM2, PACSIN3, PARD3B, PARP10, PDE4A, PNPLA3, PPP1R14A, PRKCB, PSEN1, PTMA, RAB34, RBFADN, RN7SKP151, RN7SL554P, RN7SL734P, RPS6KA6, SH3GLB2, SH3KBP1, SLC35A2, SLC35F3, SLC7A2, SRD5A3-AS1, TAF4, TMEM104, TMEM132C, TMEM132D, TMEM165, TMOD2, TNNT3, TREX2, TRIM67, TTC34, TTC40, TTLL12, USF2, VGLL4, WAS, WWTR1, WWTR1-AS1, XKR6, ZG16B, ZIK1.

List of differentially methylated genes in luminal B breast cancer samples with different response to NACT

ANK1, ARHGAP9, ARMC4P1, C1QL3, CACNA1H, CEACAM22P, CNPY1, COL18A1, COL9A1, DMRT3, EBF1, FAM83H-AS1, FBXO17, FLT4, GATA2, GNAS, HEYL, IFI27, INTS1, ISLR2, KLHL15, KRTCAP3, LAD1, LINC00354, LINC00629, LRRC37A6P, LTBR, MARS, MIR4489, NMRK2, PGR, PLXNB2, PNPLA7, RBFOX1, RFPL3, RPH3AL, RUNX3, SDK1, SIPA1, SIX1, SLC13A3, SLC16A12, SNORD111, SNTG1, TSPAN11, XKR6, ZNF578, ABCA17P, ABCA3, ABHD12B, ACADS, ADAMTS7P3, ADCY9, ADRA2A, AJAP1, ANKRD36BP2, ARID3A, ATP1A3, BARX1, BMS1P17, BNC1, BNIP3, C17orf64, CAPN15, CCDC137, CD248, CD8A, CDX1, CELF4, CKB, CLEC14A, CLEC4G, COL9A2, CRMP1, CTSA, CYBA, DKK1, DLG4, DMRTC1, DOK1, DPP6, DPYS, DPYSL3, DUSP9, EFNA2, EGFL7, EPS8L1, ERICH1, ERICH1-AS1, FAM155B, FAM228A, FAM83H-AS1, FBXL16, FOXE3, FOXH1, FOXI2, FSTL1, GALR2, GAS6, GBGT1, GLP1R, GNAT1, GPR25, GSC2, GUSBP1, HAND2, HAND2-AS1, HHAT, HMGB3, HOXD12, IBA57-AS1, IRF4, ITGB4, KLHL34, KRMEN1, L1TD1, LINC00092, LINC00159, LINC00273, LINC01044, LOXL3, LRRC38, LTBP3, MAPK12, MAPK8IP2, MIR503, MIR503HG, MIR5587, MUM1, MYO15B, NEURL2, NKX2-2, NODAL, NOTUM, NPR2, NRN1, OLIG3, OXT, PALM, PHKA2, PLCD1, PNCK, PPP1R14BP2, PPP1R16A, PPP1R16B, PRKAR1B, PRKCB, PRKG1-AS1, PROX1-AS1, PRR5, PRSS44, PRSS45, PRSS50, PSKH2, PTGIS, QRFPR, RABL6, RALGDS, RAP1GAP2, RBPJ, RHOQP2, RHOQP3, RN7SL121P, RNA5SP175, RNU6-664P, RPL10, SALL1, SEMA5B, SEPT9, SFRP2, SLC25A43, SLC30A2, SLC6A3, SLC6A8, SNAP25, SNAP25-AS1, SOX1, SOX21, SRP68, TCEA2, TERT, THNSL2, TMEM132C, TMEM132D, TMEM164, TMEM235, TMEM92, TRABD, TTC22, TTC34, USP32, VGLL4, VSX1, VWC2, WAS, WBSCR17, YBX1P1, YBX1P10, YBX1P6, ZAR1, ZIC1, ZNF630.

Table S1. Sequences and specific modifications of the sequencing adapters used in the XmaI-RRBS protocol. (M, Methylated cytosine). Unique 5 bp barcodes are highlighted in gray.

Adapter	Sequence (5′-3′)
RRBS-ADP-Ac-1	GG**TTGTTC**TGAGTCGGAGACACGCAGGGATGAGATGGTT
RRBS-ADP-A-me-1	MMATMTMATMMMTGMGTGTMTMMGAMTMAGAAMAA
RRBS-ADP-Ac-2	GG**CGATT**CTGAGTCGGAGACACGCAGGGATGAGATGGTT
RRBS-ADP-A-me-2	MMATMTMATMMMTGMGTGTMTMMGAMTMAGAATMG
RRBS-ADP-Ac-3	GG**CTTAA**CTGAGTCGGAGACACGCAGGGATGAGATGGTT
RRBS-ADP-A-me-3	MMATMTMATMMMTGMGTGTMTMMGAMTMAGTTAAG
RRBS-ADP-Ac-4	GG**CACCT**CTGAGTCGGAGACACGCAGGGATGAGATGGTT
RRBS-ADP-A-me-4	MMATMTMATMMMTGMGTGTMTMMGAMTMAGAGGTG
RRBS-ADP-Ac-5	GG**CAGTGC**TGAGTCGGAGACACGCAGGGATGAGATGGTT
RRBS-ADP-A-me-5	MMATMTMATMMMTGMGTGTMTMMGAMTMAGMAMTG
RRBS-ADP-Ac-6	GG**CGCGG**CTGAGTCGGAGACACGCAGGGATGAGATGGTT
RRBS-ADP-A-me-6	MMATMTMATMMMTGMGTGTMTMMGAMTMAGMMGMG
RRBS-ADP-Ac-7	GG**TTAGG**CTGAGTCGGAGACACGCAGGGATGAGATGGTT
RRBS-ADP-A-me-7	MMATMTMATMMMTGMGTGTMTMMGAMTMAGMMTAA
RRBS-ADP-Ac-8	GG**CCTCG**CTGAGTCGGAGACACGCAGGGATGAGATGGTT
RRBS-ADP-A-me-8	MMATMTMATMMMTGMGTGTMTMMGAMTMAGMGAGG
RRBS-ADP-Ac-9	GG**TCGAG**CTGAGTCGGAGACACGCAGGGATGAGATGGTT
RRBS-ADP-A-me-9	MMATMTMATMMMTGMGTGTMTMMGAMTMAGMTMGA
RRBS-ADP-Ac-10	GG**TATGA**CTGAGTCGGAGACACGCAGGGATGAGATGGTT
RRBS-ADP-A-me-10	MMATMTMATMMMTGMGTGTMTMMGAMTMAGTMATA
RRBS-ADP-Ac-11	GG**TCCTA**CTGAGTCGGAGACACGCAGGGATGAGATGGTT
RRBS-ADP-A-me-11	MMATMTMATMMMTGMGTGTMTMMGAMTMAGTAGGA
RRBS-ADP-Ac-12	GG**CCAGA**CTGAGTCGGAGACACGCAGGGATGAGATGGTT
RRBS-ADP-A-me-12	MMATMTMATMMMTGMGTGTMTMMGAMTMAGTMTGG
RRBS-ADP-Ac-13	GG**TGACA**CTGATCGGAGACACGCAGGGATGAGATGGTT
RRBS-ADP-A-me-13	MMATMTMATMMMTGMGTGTMTMMGAMTMAGTGTMA
RRBS-ADP-P1	GGATCACCGACTGCCCATAGAGAGGAAAGCGGAGGCGTAGTGGTT
RRBS-ADP-P1-me	MMAMTAMGMMTMMGMTTTMMTMTMTATGGGMAGTMGGTGAT

Table S2. Genome regions selected for MSRE-qPCR Positive internal Controls (PCs) and a Digestion efficacy Controls (DCs)

Target chromosome	Target start	Target end	ID
chr3	98241588	98241591	PC1_CLDND1
chr19	1154197	1154248	PC2_SBNO2
chr20	18548097	18548098	PC3_LINC00493
chr18	61670062	61670111	PC4
chr3	43663510	43663525	DC1_ANO10
chr3	43732464	43732475	DC2_ABHD5
chr3	43732768	43732797	DC3_ABHD5
chr3	45267187	45267202	DC4_TMEM158
chr3	45267747	45267774	DC5_TMEM158

Table S3. Primers and TaqMan-probes of 11 pools comprising candidate DNA methylation markers of BC NACT effectiveness. The G (general) pools include markers that discriminate both TN and luminal B tumors in terms of response to NACT; the TN (triple-negative) pools include markers exclusive for TNBC; and LB (luminal B), for luminal B subtype.

Pool_Locus	Primers sequence (5′-3′)	Length, bp	TaqMan probe sequence (5′-3′)	T, °C	BstHHI sites
G1_PC_SBNO2	F: CGTCCACTGGGGCAGCATTC<br>R: CGGAGCGAGAAGCCCAGATAGA	293	FAM-CGGGTA+AT+CC+CTGT+C+CA+TG-BHQ1		0
G1_DC_TMEM158	F: CTCCAGACCCGGTTGCGTTT<br>R: GGGAATCCTGCTCTGGGATAGCA	106	HEX-CTGCCG+CGCT+GCT+CTG-BHQ2		2
G1_TERT	F: AGAAAGGAAGGGGAGGGGCTG<br>R: CGCTGGCGTCCCTGCA	303	ROX-AGC+TG+GAA+GG+T+G+AAG-BHQ2	66	7
G1_TTC34_1	F: TCAGTGTGGCCTCTTCTGCCA<br>R: GGGAGTCTGGGGTCGGATTGA	293	CY5-TGG+T+AG+TGAAG+C+C+TC-BHQ2		3
G1_TMEM132D_3	F: GTGGCCGGGCTCGCTG<br>R: GCCGCACCCGCCAAACT	122	CY5.5-CCCCA+TCCCAGGCC+GG-BHQ2		6
G2_PC_LINC00493	F: CACAGTTCTACACCCGAAAGTCC<br>R: GTGAACTCATTTCGATACATGGGTACG	224	FAM-TCCTTAA+T+GGTTCC+GGCG-BHQ1		0
G2_DC_ANO10	F: CGGGCGAAAGAGTGCTCG<br>R: CCTGCGTGTGACCGCATC	108	HEX-AGCGCTGGGCGTGGCGGA-BHQ2	64	4
G2_TMEM132D_2	F: CCCTGCGAGCGCGGA<br>R: GCCCTTCTCCAGCCATCCTT	209	ROX-CG+T+CA+T+CAAAA+C+C+TCAG-BHQ2		4
G2_VGLL4_2	F: CTGTTCTTGGTCAGTGCGAGG<br>R: TAAATAAGCAACACGGAGTGCCTG	252	CY5-CGTTTTCT+CAAA+GG+CAAA+GGG-BHQ2		3
G3_PC3	F: CGCCCTCGGTGCCGAC<br>R: GAATGCGAGGAGAGGAGATGGAAATG	245	CY5.5-CC+GGGAT-BHQ2-AATAA+GGTCTGT+G+GGTG		0
G3_DC_ANO10	F: GGGCGAAAGAGTGCTCGGTG<br>R: CTGCGTGTGACCGCATCTAGG	106	CY5-CGGGCCAGGCCAGT-BHQ2-GGGGCGG	64	4
G3_ABCA3_1	F: GACTCCCGGGCTCCAGCA<br>R: GGCGTTGCATTAGGTCGGGG	250	FAM-ACC+ACAGT-BHQ1-GA+GGTGC+GTCCGTGGT		5
G3_DPYS_1	F: ACCCGCAGCCCCGCA<br>R: TGCAGGAGGGCACCCCAAG	241	HEX-A+GAAG+TCAT-BHQ2-CGTT+GA+CCACGC+GAC		6

Pool_Locus	Primers sequence (5′-3′)	Length, bp	TaqMan probe sequence (5′-3′)	T, °C	BstHHI sites
G3_IRF4	F: GGCAGCTCTTCTCCCCGCA<br>R: GCTCTTCTCCTCGTTCTCCCACA	219	ROX-C+CAG+T+GGCT-BHQ2-+GATCGACCAGATCG		3
G4_PC2_CLDND1	F: GAACGGCGGTTCGTCCAAG<br>R: CATGTTCCCGGCGGTTTGAAG	238	Cy5-CC+CCGGT-BHQ2-ACCC+GACCAGG		0
G4_DC6_ABHD5	F: GGCTCCCCTCAGCGTCG<br>R: GCTTATACAACAACGGGGCGG	105	FAM-CCGG+GAGGCC+GCCT-BHQ1-TGAC	62	4
G4_TMEM132C_1	F: GAGTGGCCCCGGGCAT<br>R: CGGAACCGGGAAGTTCGCA	232	HEX-AGCGGC+CGGGACGCAGG-BHQ1		9
G4_SFRP2_1	F: CAGTGCGAGGCGAGGAAGA<br>R: CAGCAACGGCTCATTCTGCT	294	ROX-CAGAG+GGAGCGGAGCCGGG-BHQ2		7
G5_PC3	F: CCCAGCCTCTCCCAGGAGGTA<br>R: TGCGAGGAGAGGAGATGGAAATGC	286	Cy5-CC+G+GGATAATAA+GGTCTG+TGG-BHQ3		0
G5_DC_ANO10	F: GGCGAAAGAGTGCTCGGTGC<br>R: CCCTGCGTGTGACCGCATC	107	FAM-CGGGCCAGGCCAGTGGGGC-BHQ1		4
G5_SOX21	F: CCCGGCCTGTGATCGCTTTC<br>R: TGCCACAGAGCTGGGCCT	150	Cy5.5-CGAGCTCCCGGGCCGGCG-BHQ3	65	4
G5_MYO15B	F: CCGGGGAGGGGAAAGGACC<br>R: CCCTGAGGCCGGCCTCC	117	HEX-CTC+CTTTGG+GCCAGCCA+TGGG-BHQ1		3
G5_TMEM132D_3	F: GGTGGCCGGGCTCGCT<br>R: CAGCCGCACCCGCCAAAC	125	ROX-CGGCCCGGGGCTCCCTGG-BHQ2		6
TN1_PC5_ALDH4A1	F: CCACTTTGATCCGACTGTG<br>R: GGAAAGAACTTCCACTTCGT	184	Cy5-TGG+CCACCGT-BHQ2-AC+TCGAAG		0
TN1_ABCA3_1	F: AGGGAGAGGTGGAGTGA<br>R: TCCAGAACATCATCAGAGTGA	384	HEX-CCAA+GAGT-BHQ2-+CCTGA+TGGAGTAG	58	5
TN1_CDO1_1	F: GATCTGTGGGGTTCATCCT<br>R: AGACAACGGGGCTCTTG	197	ROX-TTA+AG+CG+CT-BHQ2-T+GGAGTC		3
TN1_CLEC14A_1	F: CAGGGACACAACACATCG<br>R: CCGCCTCTAACTTGAGCTA	181	Cy-5.5-AGTTTGT-BHQ3-+CCAGC+GAGCG		4
TN2_PC8	F: TCCGGGAAGAACCGAAAA<br>R: CTTAAGCCACTCCCAAGC	283	Cy5.5-A+CCTC+GCT-BHQ2-CAGACTCGTG		0
TN2_DC8_TCAIM	F: GACGTCAGCTAGAGGCA<br>R: GGTGACGCCCTGGTT	108	FAM-A+CCG+CGGAAGGT-BHQ1TGAATC	58	3
TN2_DLEU2_1	F: GAGCGAAAGCAAAACGAAA<br>R: CTTTTCCAAGGGGGTTGAG	198	HEX-CCAAG+AT-BHQ2-CTG+A+GGT+CG		4

Pool_Locus	Primers sequence (5′-3′)	Length, bp	TaqMan probe sequence (5′-3′)	T, °C	BstHHI sites
TN3_PC7_LINC00493	F: GACACCTGAGCGACTTTC<br>R: GTGAACTCATTTCGATACATGG	200	Cy5.5-AAGC+GGACGC+TGAAAACT-BHQ3	58	0
TN3_DC4_ANO10	F: CGAAAGAGTGCTCGGTG<br>R: GGAGCTACCGCCCAG	153	FAM-CCTA+GAT-BHQ1-GCGGTCA+CACG		7
TN3_BNC1_1	F: CGGATAACGCCCTAAATCAG<br>R: GAGGCCGAATCATCTCCT	215	HEX-CACAGA+CGT-BHQ2-GT+CGGTGTT		3
TN3_SFRP2_1	F: AGTTCGAGCTTGTCCCG<br>R: CACCCTCCAGATTTGCATAA	232	ROX-AGA+AT+G+AGC+CGTT-BHQ2-GCT		4
TN3_TTC34_1	F: ATTGTTGGACCTGGGGT<br>R: CTTGATCTCCCCTCTTGGT	230	Cy-5-CGCCAT-BHQ2-GAGC+TC+TG+AGTC		3
TN4_PC6	F: TAGAGGAAGTCGTAGAGGTGT<br>R: TCATTGTGTCTTGACAACCG	244	Cy5.5-CGG+CACT-BHQ2-AG+CAGAGACCA	58	0
TN4_DC10_TMEM158	F: CCCCAGGTGCTCGATG<br>R: CGACCTACTGCTCTTCTCC	97	FAM-CGAAGA+AAGCGCGGCCGt-BHQ1		2
TN4_PRKCB_2	F: TCAAGAACCACAAATTCACCG<br>R: ACTGTCCATCCGGGAGT	240	HEX-CTT+CA+TCT-BHQ2-+GGT+GAGCG		6
TN4_GMDS_1	F: CAGCTCCCCTCACTTCTC<br>R: TCGCTTTCGATGTGAGTATCT	148	Cy5-CCCG+ACCC+TGAG+AGC-BHQ3		3
TN6_PC10_FAM83A	F: CACCTCTACGCCTCCTCCAA<br>R: TGAAGACGCGGACACACTTC	198	Cy5.5-AAT+GGCC+GCCT-BHQ2-TA+GCAGC	62	0
TN6_DC10_TMEM158	F: GGTGCTCGATGAGCAGCG<br>R: CTTCCAGTGCGACCTACTGC	101	FAM-CGAA+GAAAGCGCG+GCCGT-BHQ1		2
TN6_MYO15B_1	F: TGCACTGCACAGAAGGTCAC<br>R: CGACTCCTGCTCCCCTGA	166	ROX-CGG+GA+G+G+CCAA+ATCC-BHQ2		3
TN6_TMEM132D_1	F: AAAAGCCCCACCCTTTCGG<br>R: CTCAGTGTGGCGTGTCAGAG	373	Cy5-CAC+CGGCCT+CT-BHQ2-CGTC+GTC		3
LB1_PC3	F: TCTCCCAGGAGGTAGGGAC<br>R: CACAGACCTTATTATCCCGGC	174	Cy5.5-AAGGCAGAA+GGCCCCCAA-BHQ3	62	0
LB1_DC12_TMEM158	F: GACCCGGTTGCGTTTGG<br>R: GGGAATCCTGCTCTGGGATA	101	FAM-CTGC+CGCGCT-BHQ1-GCTCTG		3
LB1_LTBR	F: GAAAAACTCCCACAGTAGGGC<br>R: GAGCAGAGGGAGTTCCAGAG	198	ROX-TTCTGC+GGCC+TT-BHQ2-GCAGTC		3
LB1_NRN1	F: GCTGTGGCCATCTCTTTCC<br>R: CGGGACACATTCACACACAA	230	Cy-5-CT+GGAAGC+TG+AGTGCC-BHQ3		5

Table S4. Predictive value of individual epigenetic markers for predicting sensitivity to NACT for triple-negative breast tumors, measured by MSRE-qPCR, *n*=48

Target locus	Sensitive tumors group b-value	Resistant tumors group b-value	Sensitiv-ity	Specific-ity	Accu-racy	cvAUC	95% CI	p-value
G2_TMEM132D_2	59.46	79	0.64	0.72	0.66	0.72	0.71-0.74	0.01
G3_ABCA3_1	36.01	49.09	0.8	0.6	0.74	0.69	0.67-0.70	0.02
G3_DPYS_1	33.72	57.27	0.64	0.66	0.64	0.67	0.65-0.69	0.01
G5_MYO15B	43.35	58.28	0.58	0.66	0.60	0.65	0.64-0.67	0.03
TN4_GMDS_1	60.93	73.16	0.73	0.71	0.72	0.64	0.63-0.66	0.06
TN1_CDO1_1	38.46	53.94	0.77	0.58	0.71	0.64	0.62-0.66	0.06
TN3_SFRP2_1	23.8	37.63	0.58	0.64	0.60	0.63	0.62-0.65	0.03
TN2_DLEU2_1	50.82	67.88	0.78	0.53	0.70	0.63	0.61-0.64	0.07
G4_SFRP2_1	16.36	28.05	0.72	0.58	0.67	0.62	0.60-0.64	0.10
G3_IRF4	26.01	36.4	0.65	0.57	0.63	0.6	0.58-0.62	0.12
G4_TMEM132C_1	12.06	29.58	0.72	0.54	0.67	0.6	0.58-0.62	0.11
G2_VGLL4_2	97.16	98.7	0.49	0.73	0.57	0.59	0.57-0.61	0.22
G1_TERT	37.95	51.83	0.65	0.63	0.64	0.58	0.56-0.59	0.19
G5_TMEM132D_3	40.52	49.48	0.43	0.74	0.53	0.57	0.56-0.59	0.16
G5_SOX21	43.47	53.85	0.69	0.48	0.63	0.57	0.55-0.59	0.16
TN1_CLEC14A_1	78.42	83.8	0.46	0.7	0.53	0.51	0.49-0.52	0.50
G1_TMEM132D_3	36.09	42.97	0.6	0.48	0.57	0.5	0.48-0.52	0.63
TN4_PRKCB_2	22.71	29.63	0.78	0.25	0.61	0.49	0.47-0.51	0.24
LB1_LTBR	55.27	61.38	0.75	0.25	0.59	0.46	0.44-0.48	0.69
G1_TTC34_1	29.82	30.78	0.02	0.98	0.32	0.43	0.41-0.44	0.47
TN1_ABCA3_1	38.33	39.75	0	1	0.31	0.38	0.36-0.40	0.83
TN3_TTC34_1	47.63	48.49	0	1	0.31	0.36	0.35-0.38	0.90
TN3_BNC1_1	58.72	60.51	0	1	0.31	0.36	0.35-0.38	0.85
LB1_NRN1	17.98	18.46	0	1	0.31	0.35	0.33-0.37	0.88

Table S5. Predictive value of individual epigenetic markers for predicting sensitivity to NACT for luminal B breast tumors, measured by MSRE-qPCR, *n*=35

Target locus	Sensitive tumors group b-value	Resistant tumors group b-value	Sensitivity	Specificity	Accuracy	cvAUC	95% CI	p-value
LB1_LTBR	51.24	58.76	0.66	0.75	0.70	0.69	0.67-0.71	0.02
G2_VGLL4_2	90.89	94.83	0.78	0.58	0.69	0.63	0.62-0.65	0.07
G3_DPYS_1	42.8	29.21	0.61	0.52	0.57	0.6	0.58-0.62	0.15
TN1_CLEC14A_1	84.38	93.17	0.34	0.9	0.59	0.57	0.55-0.59	0.19
G1_TTC34_1	23.72	28.36	0.47	0.67	0.57	0.56	0.54-0.58	0.23
TN3_BNC1_1	63.98	72.78	0.76	0.39	0.59	0.54	0.52-0.56	0.35
TN4_SMDS_1	81.16	79.03	0.63	0.43	0.54	0.5	0.48-0.52	0.32
TN1_ABCA3_1	47.94	42.08	0.42	0.68	0.54	0.5	0.48-0.52	0.42
TN3_TTC34_1	57.41	52.17	0.39	0.7	0.53	0.49	0.48-0.51	0.64
TN3_SFRP2_1	53.6	60.79	0.29	0.82	0.53	0.47	0.45-0.49	0.84
G3_IRF4	48.47	44.06	0.3	0.73	0.50	0.44	0.43-0.46	0.57
G3_ABCA3_1	41.53	38.04	0	1	0.46	0.43	0.41-0.45	0.64
G4_TMEM132C_1	25.94	20.03	0.21	0.81	0.49	0.43	0.41-0.45	0.99
LB1_NRN1	38.34	33.5	0.16	0.88	0.49	0.42	0.40-0.43	0.73
G1_TMEM132D_3	39.91	42.23	0	1	0.46	0.4	0.38-0.41	0.78
G4_SFRP2_1	29.36	32.01	0	1	0.46	0.37	0.35-0.39	0.72
TN1_CDO1_1	65.4	63.22	0	1	0.46	0.36	0.35-0.38	0.48
G5_TMEM132D_3	36.41	38.84	0	1	0.46	0.36	0.34-0.38	0.79
G5_MYO15B	66.43	65.25	0	1	0.46	0.35	0.33-0.37	0.73
G5_SOX21	60.51	59.56	0	1	0.46	0.34	0.33-0.36	0.59
G2_TMEM132D_2	54.13	53.15	0	1	0.46	0.33	0.32-0.35	0.99
G1_TERT	31.54	30.32	0	1	0.46	0.32	0.30-0.34	0.93
TN2_DLEU2_1	64.51	64.05	0	1	0.46	0.32	0.30-0.34	0.87
TN4_PRKCB_2	43.46	42.71	0	1	0.46	0.32	0.30-0.33	0.83

Table S6. Predictive value of top 10 panels of epigenetic markers for predicting sensitivity to NACT of triple-negative breast tumors, measured by MSRE-qPCR.

Target locus	Sensitivity	Specificity	Accuracy	cvAUC	95% CI
G2_TMEM132D_2, G5_MYO15B	0.76	0.76	0.76	0.83	0.81-0.83
G2_TMEM132D_2, G4_TMEM132C_1, G5_MYO15B	0.76	0.76	0.76	0.83	0.81-0.83
G2_TMEM132D_2, G5_MYO15B, TN2_DLEU2_1	0.66	0.88	0.73	0.82	0.81-0.83
G2_TMEM132D_2, G5_MYO15B, TN3_SFRP2_1	0.72	0.77	0.74	0.82	0.81-0.83
G2_TMEM132D_2, G2_VGLL4_2, G4_TMEM132C_1, G5_MYO15B	0.63	0.85	0.70	0.82	0.80-0.82
G2_TMEM132D_2, G5_MYO15B, TN3_SFRP2_1, TN4_GMDS_1	0.52	0.94	0.65	0.82	0.80-0.82
G2_TMEM132D_2, G3_IRF4, G5_MYO15B	0.75	0.75	0.75	0.82	0.80-0.82
G2_TMEM132D_2, G3_ABCA3_1, G5_MYO15B, TN3_SFRP2_1	0.71	0.76	0.73	0.81	0.80-0.82
G2_TMEM132D_2, G3_ABCA3_1, G5_MYO15B	0.78	0.70	0.75	0.81	0.80-0.82
G2_TMEM132D_2, G4_TMEM132C_1, G5_MYO15B, TN2_DLEU2_1	0.58	0.89	0.68	0.81	0.80-0.82

Table S7. Predictive value of top 10 panels of epigenetic markers for predicting sensitivity to NACT of luminal B breast tumors, measured by MSRE-qPCR.

Target locus	Sensitivity	Specificity	Accuracy	cvAUC	95% CI
G1_TTC34_1, LB1_LTBR, TN1_CLEC14A_1	0.70	0.79	0.74	0.76	0.74-0.77
LB1_LTBR, TN1_CLEC14A_1	0.83	0.69	0.77	0.75	0.73-0.76
G1_TTC34_1, G3_DPYS_1, LB1_LTBR, TN1_CLEC14A_1	0.64	0.78	0.71	0.74	0.72-0.75
G3_DPYS_1, LB1_LTBR, TN1_CLEC14A_1	0.83	0.63	0.74	0.74	0.72-0.75
G1_TTC34_1, G2_VGLL4_2, LB1_LTBR, TN1_CLEC14A_1	0.69	0.77	0.73	0.72	0.70-0.74
G2_VGLL4_2, LB1_LTBR, TN1_CLEC14A_1	0.81	0.67	0.74	0.72	0.70-0.73
G2_VGLL4_2_Cy5, G3_DPYS_1, LB1_LTBR, TN1_CLEC14A_1	0.70	0.73	0.71	0.71	0.69-0.73
G2_VGLL4_2, G3_DPYS_1, TN1_CLEC14A_1	0.72	0.70	0.71	0.71	0.68-0.72
G1_TTC34_1, G2_VGLL4_2, G3_DPYS_1, LB1_LTBR, TN1_CLEC14A_1	0.62	0.77	0.69	0.71	0.68-0.71
G1_TTC34_1, G3_DPYS_1, TN1_CLEC14A_1	0.75	0.66	0.71	0.70	0.68-0.69

Table S8. Predictive value of top 10 combined panels of epigenetic and clinical/morphological markers for predicting NACT sensitivity of triple-negative breast tumors.

Target locus	Sensitivity	Specificity	Accuracy	cvAUC	95% CI
G2_TMEM132D_2, G4_TMEM132C_1, G5_MYO15B, S	0.76	0.80	0.79	0.87	0.85-0.88
G2_TMEM132D_2, G5_MYO15B, DLEU2, S	0.80	0.80	0.82	0.86	0.85-0.87
G2_TMEM132D_2, G2_VGLL4_2, G4_TMEM132C_1, G5_MYO15B, S	0.81	0.77	0.80	0.86	0.84-0.87
G2_TMEM132D_2, G5_MYO15B, S	0.84	0.71	0.79	0.86	0.84-0.87
G2_TMEM132D_2, TN3_SFRP2_1, G5_MYO15B, S	0.85	0.76	0.83	0.86	0.84-0.87
G2_TMEM132D_2, G5_MYO15B, SFRP2, S	0.79	0.79	0.81	0.86	0.84-0.86
G2_TMEM132D_2, TN3_SFRP2_1, G5_MYO15B, S	0.89	0.68	0.81	0.86	0.84-0.86
TERT, G2_TMEM132D_2, G4_TMEM132C_1, TN3_SFRP2_1, S	0.76	0.85	0.83	0.85	0.84-0.86
G2_TMEM132D_2, G3_DPYS_1, G5_MYO15B, TN2_DLEU2_1, S	0.80	0.80	0.84	0.85	0.84-0.86
G2_TMEM132D_2, G4_TMEM132C_1, G5_MYO15B, TN3_SFRP2_1, S	0.82	0.78	0.80	0.85	0.84-0.86

Table S9. Predictive value of top 10 combined panels of epigenetic and clinical/morphological markers for predicting NACT sensitivity of luminal B breast tumors.

Target locus	Sensitivity	Specificity	Accuracy	cvAUC	95% CI
LB1_LTBR, TN1_CLEC14A_1, N	0.89	0.71	0.81	0.83	0.82-0.85
G3_DPYS_1, N	0.67	0.91	0.78	0.83	0.81-0.84
G3_DPYS_1, TN1_CLEC14A_1, N	0.86	0.74	0.80	0.82	0.81-0.84
G2_VGLL4_2, G3_DPYS_1, TN1_CLEC14A_1	0.79	0.79	0.79	0.82	0.80-0.83
G2_VGLL4_2, G3_DPYS_1, N	0.73	0.84	0.78	0.82	0.79-0.82
G3_DPYS_1, LB1_LTBR, TN1_CLEC14A_1, N	0.81	0.75	0.79	0.81	0.79-0.82
G2_VGLL4_2, LB1_LTBR, TN1_CLEC14A_1, N	0.84	0.72	0.79	0.81	0.79-0.82
TN3_TTC34_1, G3_DPYS_1, N	0.68	0.85	0.76	0.81	0.79-0.82
TN3_TTC34_1, LB1_LTBR, TN1_CLEC14A_1, N	0.81	0.81	0.81	0.81	0.78-0.81
TN3_TTC34_1, TN1_CLEC14A_1, N	0.77	0.77	0.77	0.81	0.79-0.82

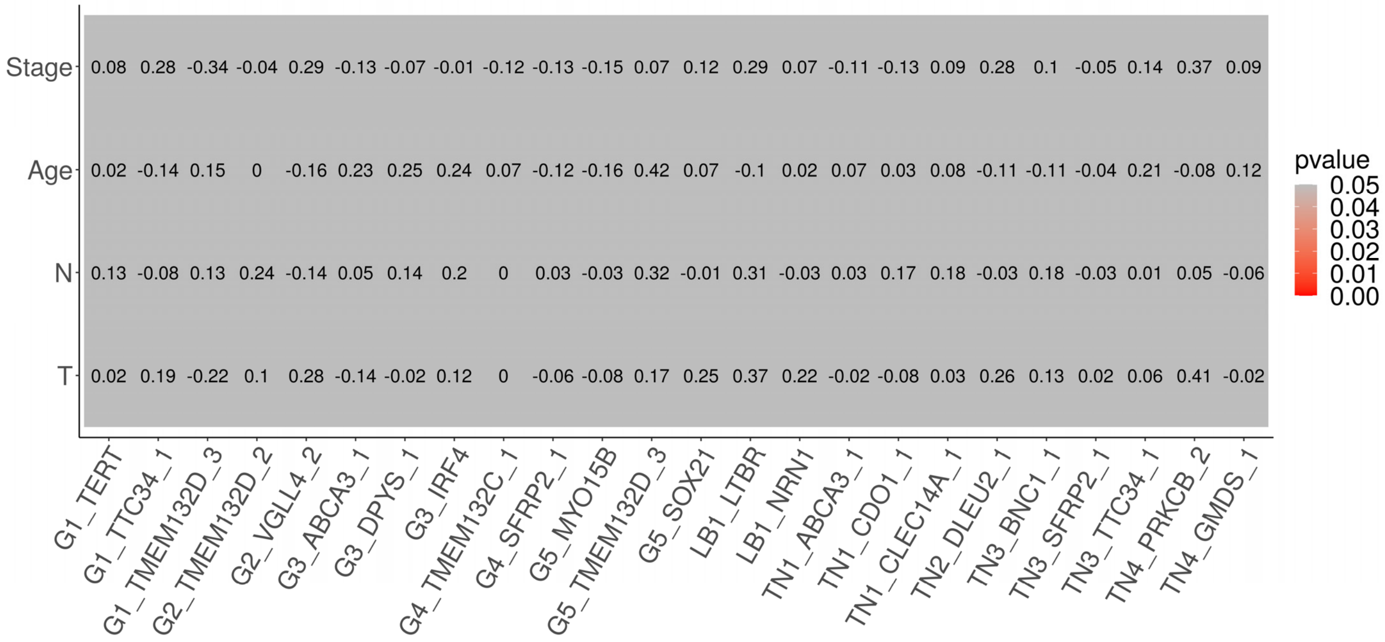

Figure S1. Correlations between clinical characteristics and the level of methylation of the selected markers in triple-negative breast cancer. Numbers show the correlation coefficient. No significant (p<0.05) correlations found, *n*=48.

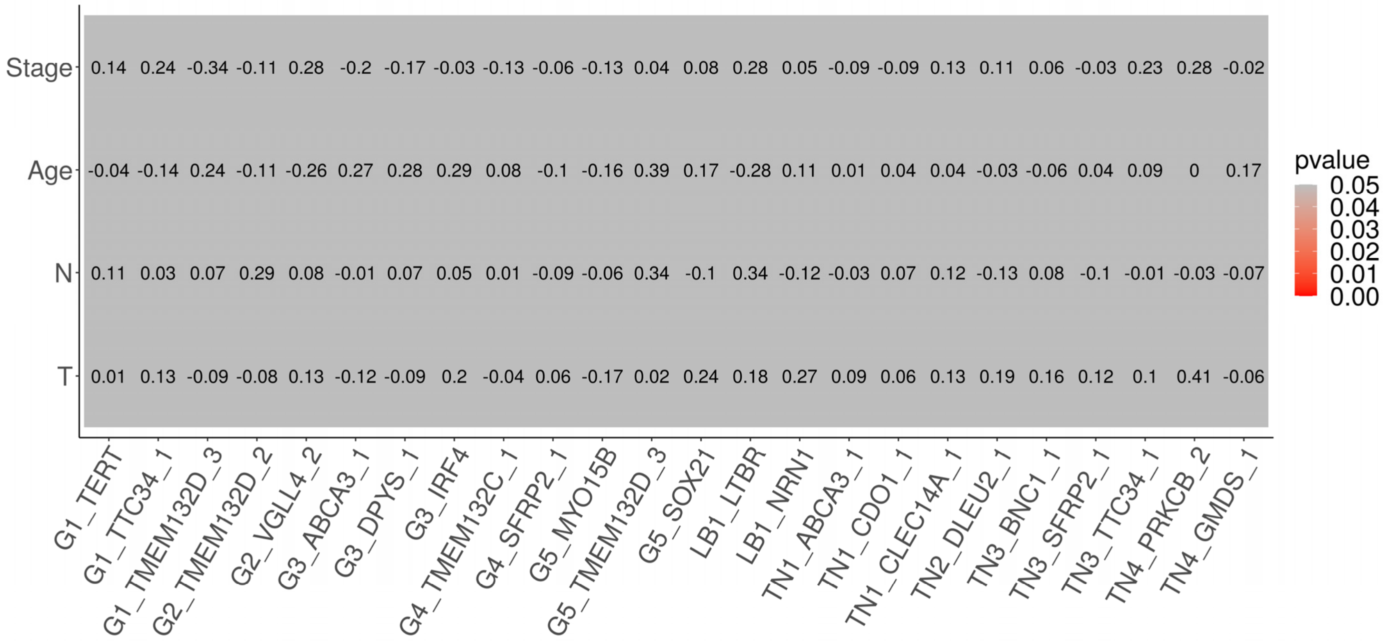

Figure S2. Correlations between clinical characteristics and the level of methylation of the selected markers in luminal B breast cancer. Numbers show the correlation coefficient. No significant (p<0.05) correlations found, *n*=35.

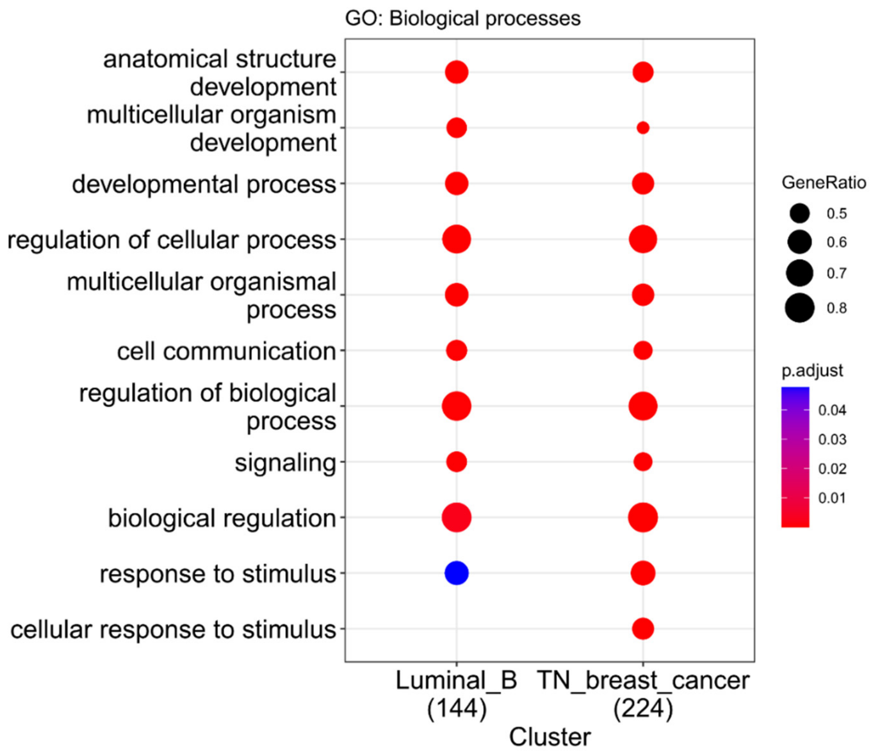

Figure S3. Top-10 over-represented Gene Ontology (GO) Biological Processes (BP) terms in TN and luminal B breast cancer subtypes. Y-axis stands for GO:BP terms, X-axis represents breast cancer subtypes, size of bubbles reflects gene ratio (genes in input/all genes in term).

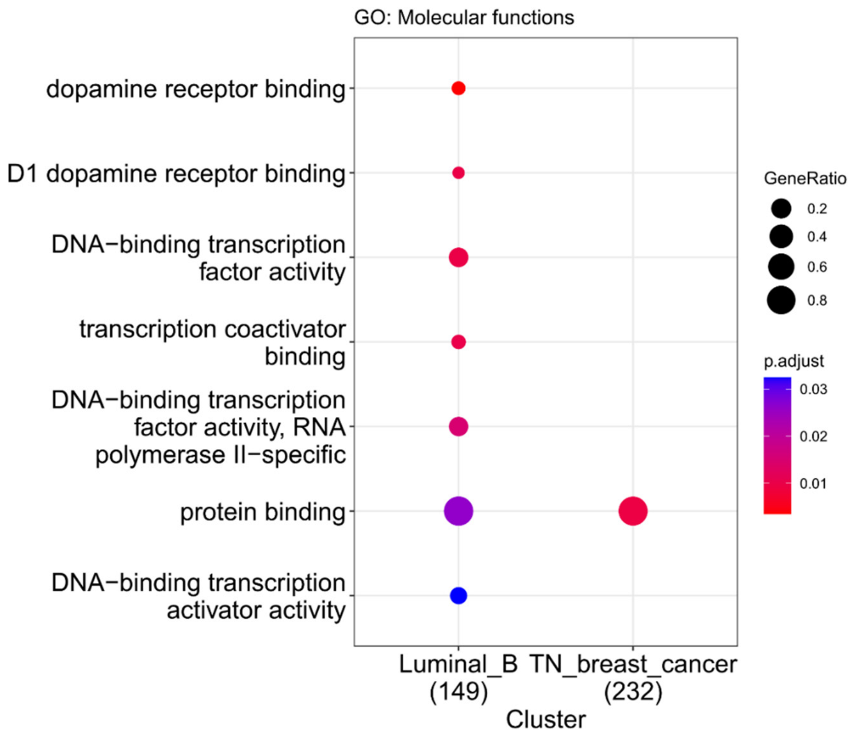

Figure S4. Top-10 over-represented Gene Ontology (GO) Molecular functions (MF) terms in TN and luminal B breast cancer subtypes. Y-axis stands for GO:MF terms, X-axis represents breast cancer subtypes, size of bubbles reflects gene ratio (genes in input/all genes in term).

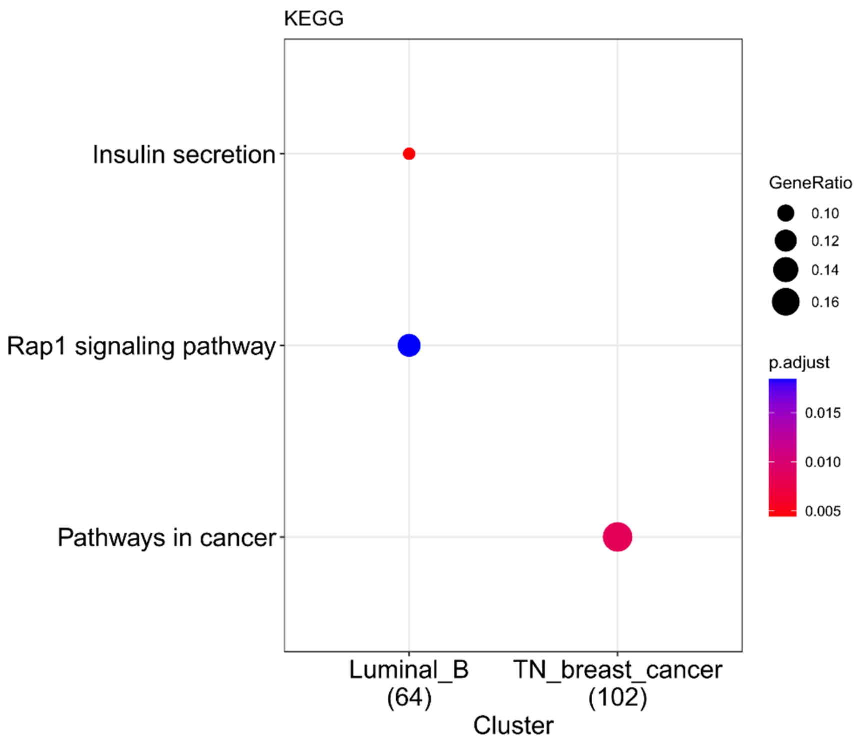

Figure S5. Top-10 over-represented Kyoto Encyclopedia of Genes and Genomes (KEGG) terms in TN and luminal B breast cancer subtypes. Y-axis stands for KEGG terms, X-axis represents breast cancer subtypes, size of bubbles reflects gene ratio (genes in input/all genes in term).

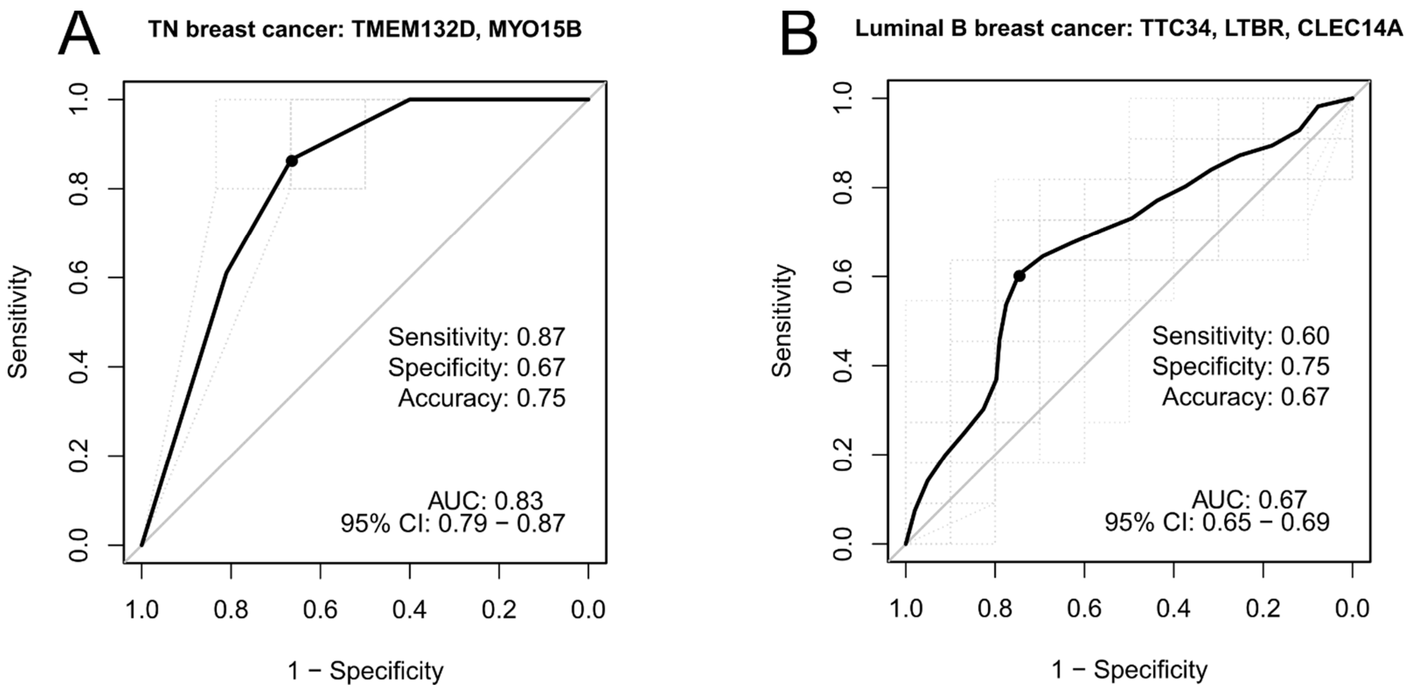

Figure S6. ROC curves, cross-validated area under the curve (cvAUC) for breast cancer NACT sensitivity classifiers for TN (A) and luminal.B subtypes (B), validated using discovery cohort RRBS data.
